# Camera-Based Monocular Depth Estimation in Orthodontics: Vision Transformer vs. CNN Model Performance

**DOI:** 10.3390/s25216512

**Published:** 2025-10-22

**Authors:** Arda Arısan, Gökhan Serhat Duran

**Affiliations:** 1 Independent Researcher, Ankara 06000, Turkey; 2 Department of Orthodontics, Faculty of Dentistry, Çanakkale Onsekiz Mart University, Çanakkale 17100, Turkey; gokhanserhat.duran@comu.edu.tr

**Keywords:** Monocular Depth Estimation, vision transformer, Convolutional Neural Networks, computer vision, medical imaging, orthodontic diagnostics

## Abstract

Background: Monocular Depth Estimation (MDE) is a computer vision approach that predicts spatial depth information from a single two-dimensional image. In orthodontics, where facial soft-tissue evaluation is integral to diagnosis and treatment planning, such methods offer new possibilities for obtaining sagittal profile information from standard frontal photographs. This study aimed to determine whether MDE can extract clinically meaningful information for facial profile assessment. Methods: Standardized frontal photographs and lateral cephalometric radiographs from 82 adult patients (48 Class I, 28 Class II, 6 Class III) were retrospectively analyzed. Three clinically relevant soft-tissue landmarks—Upper Lip Anterior (ULA), Lower Lip Anterior (LLA), and Soft Tissue Pogonion (Pog′)—were annotated on frontal photographs, while true vertical line (TVL) analysis from cephalograms served as the reference standard. For each case, anteroposterior (AP) relationships among the three landmarks were represented as ordinal rankings based on predicted depth values, and accuracy was defined as complete agreement between model-derived and reference rankings. Depth maps were generated using one vision transformer model (DPT-Large) and two CNN-based models (DepthAnything-v2 and ZoeDepth). Model performance was evaluated using accuracy, 95% confidence intervals, and effect size measures. Results: The transformer-based DPT-Large achieved clinically acceptable accuracy in 92.7% of cases (76/82; 95% CI: 84.8–97.3), significantly outperforming the CNN-based models DepthAnything-v2 (9.8%) and ZoeDepth (4.9%), both of which performed below the theoretical chance level (16.7%). Conclusions: Vision transformer-based Monocular Depth Estimation demonstrates the potential for clinically meaningful soft-tissue profiling from frontal photographs, suggesting that depth information derived from two-dimensional images may serve as a supportive tool for facial profile evaluation. These findings provide a foundation for future studies exploring the integration of depth-based analysis into digital orthodontic diagnostics.

## 1. Introduction

The primary objective of orthodontic treatment is to achieve both functional occlusion and an esthetic facial appearance. In this context, soft tissue profile analysis has become an indispensable component of orthodontic planning. For many years, traditional approaches focused primarily on skeletal and dental structures. However, the importance of soft tissue evaluation has gained increasing recognition in recent decades. This shift is closely linked to rising esthetic expectations and a better understanding of the influence of soft tissue morphology on treatment outcomes [1].

Soft tissue analysis plays a critical role in orthodontic and dentofacial orthopedic treatment planning, helping predict post-treatment esthetics, improve facial balance, and enhance patient satisfaction [2]. Given that malocclusion is among the most prevalent oral health conditions globally—affecting nearly one-third of the population—the demand for efficient, accurate, and esthetically driven diagnostic tools has become increasingly important [3]. Since the 1930s, lateral cephalometric radiographs have served as the standard diagnostic tool for evaluating these relationships, providing essential information for both hard- and soft-tissue assessment [4]. In contemporary orthodontics, cephalometry remains the gold standard for evaluating sagittal skeletal relationships and guiding a wide range of clinical decisions, including extraction versus non-extraction therapy, growth modification, and orthognathic surgical planning. Quantitative measurements such as the ANB angle, Wits appraisal, facial convexity, and Holdaway ratio provide critical diagnostic data for assessing anteroposterior jaw relationships and lip–chin balance. These parameters enable clinicians to predict treatment outcomes, plan biomechanical approaches, and assess facial harmony. Although photographic and 3D imaging techniques have advanced in recent decades, none have achieved the same level of diagnostic reproducibility, standardization, and prognostic reliability as lateral cephalometry [5,6].

To complement traditional radiographic evaluation, three-dimensional facial scanning technologies integrated with Artificial Intelligence have been developed. Techniques such as stereophotogrammetry, structured light projection, and laser scanning can capture soft tissue morphology with high resolution and without radiation exposure [7]. Compared with two-dimensional methods, these systems provide more comprehensive and accurate data for assessing facial geometry [8]. However, 3D scanners have notable limitations. They require costly equipment, controlled environments, and trained personnel, restricting their routine use in clinical settings. Additionally, movement artifacts, lighting variations, and calibration issues can compromise reproducibility and reliability. Consequently, despite their diagnostic benefits, these systems are less feasible for regular use—especially in children or large-scale applications—due to their high cost and technical complexity [9].

Recent advances in Monocular Depth Estimation (MDE) and 2D-to-3D facial reconstruction have enabled the generation of depth maps from standard frontal photographs, offering new avenues for noninvasive soft-tissue analysis. In dermatology, depth mapping has been successfully applied in lesion assessment and chronic wound monitoring [10,11]. Similarly, in otolaryngology, monocular endoscopic imaging combined with Deep Learning has shown promise in extracting depth information during minimally invasive procedures [12,13].

Unlike conventional 3D facial scanning methods that rely on specialized hardware, controlled environments, and multiple viewpoints or structured light projection, Monocular Depth Estimation (MDE) operates on a fundamentally different principle. It infers spatial depth information from a single standard two-dimensional photograph using Deep Learning models trained to recognize visual cues such as perspective, occlusion, shading, and texture gradients. This approach eliminates the need for costly equipment, calibration procedures, or operator expertise, making it inherently more accessible for routine clinical use, large-scale screening, and retrospective analysis of existing photographic records.

Building on these developments, the present study explores whether Monocular Depth Estimation can extract clinically meaningful anteroposterior information from standard frontal facial photographs. The aim is to determine whether depth cues derived from two-dimensional images can approximate sagittal soft-tissue relationships that are traditionally assessed on lateral cephalograms. By comparing the output of advanced Deep Learning models with reference cephalometric data, this work provides an initial step toward evaluating the potential of Monocular Depth Estimation as a complementary diagnostic approach for soft-tissue assessment in orthodontics.

## 2. Materials and Methods

**Figure 1:**
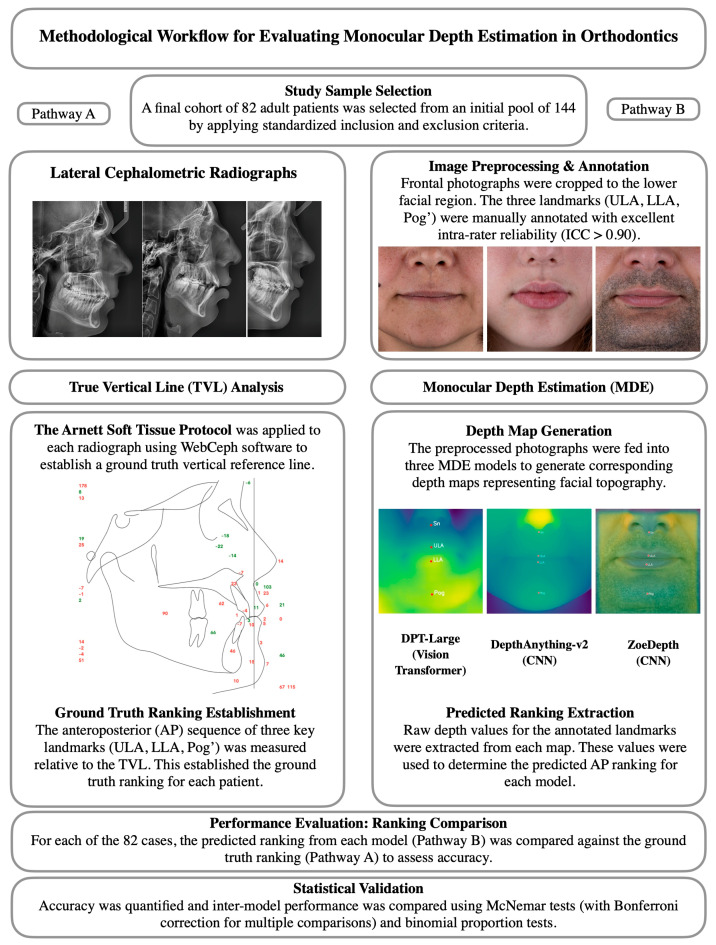
Methodological workflow of the study. The left pathway (**A**) illustrates the ground-truth derivation from lateral cephalometric radiographs using the TVL analysis, while the right pathway (**B**) shows the Monocular Depth Estimation process from frontal photographs and subsequent ranking comparison.

The following section describes the methodological framework of this study, including participant selection, imaging procedures, data preprocessing, depth estimation models, and statistical analysis, as summarized in Figure 1.

### 2.1. Study Design and Sample Size Determination

An a priori power analysis was performed using a paired proportions framework (McNemar test). Following established methodologies for facial measurement accuracy studies [14,15], and assuming discordant proportions of 0.10 and 0.40 between model predictions and the cephalometric reference standard, a minimum sample size of 66 patients was required to achieve 95% statistical power at a two-tailed alpha level of 0.05. The corresponding odds ratio (OR = 4.0) represented a large effect size, confirming adequate sensitivity to detect statistically significant differences.

This retrospective study initially enrolled 144 adult patients (89 Class I, 44 Class II, 11 Class III) from a single private orthodontic practice. Data were collected between 2023 and 2024, following written informed consent. For each patient, standardized frontal and profile photographs, together with lateral cephalometric radiographs, were obtained for subsequent analysis.

### 2.2. Image Acquisition and Standardization

Patients were eligible for inclusion if they were 18 years of age or older, had high-resolution frontal photographs captured under standardized studio conditions using a Canon EOS 5D Mark IV DSLR camera (30.4 MP full-frame CMOS sensor; Canon Inc., Tokyo, Japan) equipped with a Canon 85 mm f/1.2 L lens (Canon Inc., Tokyo, Japan) and a paraflash system to eliminate shadows and specular highlights, and possessed calibrated lateral cephalometric radiographs in TIFF format. The camera was mounted on a tripod at a fixed distance of 1.5 m from the patient, with the lens positioned at the level of the nasion to maintain consistent perspective across all photographs. Illumination was provided by an Elinchrom BRX 500/500 Two-Light Softbox Kit (Elinchrom S.A., Renens, Switzerland) arranged symmetrically on both sides of the camera to ensure uniform, shadow-free lighting and reproducible exposure conditions. Frontal photographs, obtained as direct outputs of a professional camera sensor, ensured consistent image quality suitable for computer vision–based analysis. Patients were excluded if they wore accessories such as glasses or hats during photography, had facial hair or hairstyles that obscured the soft tissue profile, exhibited improper head alignment (i.e., not parallel to the Camper’s plane), or presented with severe skeletal facial asymmetry, as determined by clinical and radiographic evaluation. After applying these inclusion and exclusion criteria, a total of 82 patients were retained for final analysis. This final cohort consisted of 48 Class I, 28 Class II, and 6 Class III individuals, representing a distribution consistent with global malocclusion prevalence [16].

### 2.3. Reference Standard: True Vertical Line (TVL) Analysis

To establish a reference standard for evaluating depth-based estimations, a True Vertical Line (TVL) analysis was performed on lateral cephalometric radiographs using the WebCeph software (version 1.0.3, 2024; WebCeph Co., Seoul, Republic of Korea). The TVL was defined as a vertical reference line passing through the subnasale point, a standard craniofacial landmark used for sagittal profile assessment according to the Arnett soft-tissue analysis protocol [17,18]. All cephalometric tracings were manually verified and fine-tuned by an experienced orthodontist to ensure landmark precision and alignment consistency across the dataset. The analysis focused on three clinically relevant soft-tissue landmarks: the Upper Lip Anterior (ULA), representing the most prominent point of the upper lip; the Lower Lip Anterior (LLA), indicating the most forward point of the lower lip; and the Soft Tissue Pogonion (Pog′), defined as the most anterior point on the chin’s soft-tissue contour.

**Figure 2:**
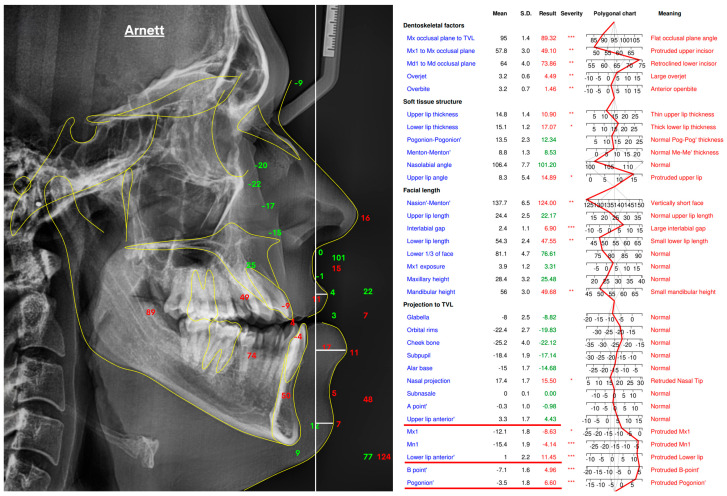
True Vertical Line (TVL)–based soft-tissue analysis performed in WebCeph (v1.0.3) following the Arnett protocol, illustrating the lateral cephalometric tracing and corresponding landmark projections in millimeters relative to the TVL, which served as the ground-truth reference for depth estimation. Asterisks indicate severity levels automatically assigned by the WebCeph software (* mild, ** moderate, *** severe).

For each patient, the anteroposterior (AP) positions of these landmarks were measured relative to the subnasale-based TVL, and their sequential AP order was recorded. This ranking served as the ground-truth reference for evaluating the accuracy of Monocular Depth Estimation models. Representative examples of the TVL analysis and corresponding numeric projections are shown in Figure 2.

### 2.4. Landmark Annotation and Data Preprocessing

To prepare the data for depth-based analysis, the lower facial height region was first isolated from the full-frontal photographs to generate anatomically focused input images. Within this cropped region, three clinically relevant soft-tissue landmarks—Upper Lip Anterior (ULA), Lower Lip Anterior (LLA), and Soft Tissue Pogonion (Pog′)—were manually annotated. All annotations were performed by a single experienced orthodontist at two time points, spaced two weeks apart, using a custom-built Python-based graphical interface designed for reproducible image annotation. These reference coordinates were established prior to depth estimation and served as anchor points for subsequent alignment with the predicted pixel-level depth values, thereby creating standardized pre-processed inputs for computer vision analysis.

The (x, y) coordinates of each landmark were recorded at both time points, and intra-observer reliability was assessed using Intraclass Correlation Coefficients (ICCs), calculated separately for each axis. A two-way mixed-effects model with absolute agreement (single measures) was used for the ICC analysis. Final reference coordinates were calculated as the arithmetic mean of the two annotations. All coordinate data were stored in structured JSON format to ensure reproducibility, consistency, and seamless integration with downstream computer vision and morphometric analysis workflows.

### 2.5. Depth Estimation Models and Implementation

Depth estimation was conducted using three state-of-the-art Monocular Depth Estimation (MDE) models: DPT-Large (vision transformer architecture; Intel Intelligent Systems Lab, Munich, Germany), DepthAnything-v2 (CNN-based, Hong Kong University of Science and Technology, Hong Kong, China), and ZoeDepth (CNN-based, ETH Zurich, Zurich, Switzerland, and Microsoft Research, Redmond, WA, USA). All models were implemented in Python (version 3.10; Python Software Foundation, Wilmington, DE, USA) with PyTorch (Meta Platforms Inc., Menlo Park, CA, USA) on Google Colab (Google LLC, Mountain View, CA, USA), accelerated by an NVIDIA Tesla T4 GPU (16 GB VRAM; NVIDIA Corporation, Santa Clara, CA, USA), a commonly used cloud-based processor for Deep Learning workflows. Pre-trained model weights were obtained from official repositories and used without additional fine-tuning to ensure full reproducibility. Model specifications are summarized in Table 1.

All models were implemented using their official pre-trained weights without additional fine-tuning or hyperparameter modification. Inference was performed at the native input resolutions specified above, using the default preprocessing and postprocessing pipelines provided by the respective frameworks. Depth maps were generated directly from the raw model outputs without normalization or scaling to preserve the original depth distributions predicted by each architecture. These raw depth values were subsequently sampled at the annotated landmark coordinates (Upper Lip Anterior, Lower Lip Anterior, and Soft Tissue Pogonion) for quantitative comparison with reference cephalometric data.

**Figure 3:**
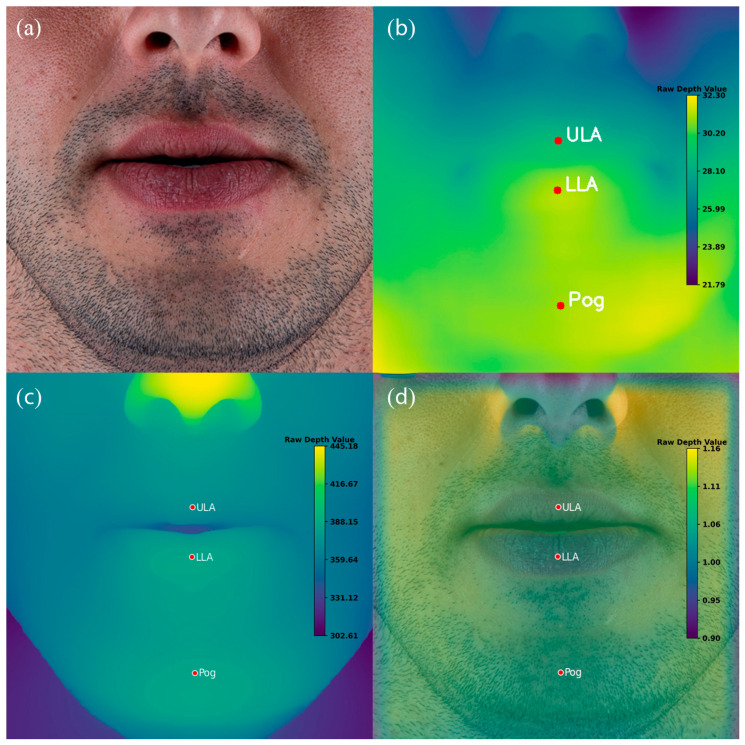
Example of Monocular Depth Estimation (MDE) outputs for a representative patient. (**a**) Original standardized frontal photograph. (**b**) Depth map generated by the vision transformer–based model (DPT-Large). (**c**) Depth map generated by the CNN-based model DepthAnything-v2. (**d**) Depth map generated by the CNN-based model ZoeDepth. Red markers indicate manually annotated landmarks: Upper Lip Anterior (ULA), Lower Lip Anterior (LLA), and Soft Tissue Pogonion (Pog′). Note that the color scales vary across models and are not directly comparable.

For each patient, two outputs were generated by each of the three depth estimation models. First, a visual depth map was produced, providing a color-coded representation of the facial surface topography. Second, quantitative depth values were extracted for the three annotated landmarks—Upper Lip Anterior (ULA), Lower Lip Anterior (LLA), and Soft Tissue Pogonion (Pog′). These values, expressed as raw unitless depth scores, were obtained directly from the corresponding depth maps. Using these measurements, the predicted anteroposterior rank order of the three landmarks was calculated for each model and patient (Figure 3).

### 2.6. Statistical Analysis

A comprehensive statistical framework was employed to evaluate the ranking accuracy and comparative performance of the three depth estimation models. The analysis included descriptive statistics, confidence interval estimation, pairwise model comparisons, and subgroup analysis across different levels of ranking complexity. All procedures were conducted using Jamovi (Version 2.6.26.0; The Jamovi Project, Sydney, Australia, 2024), running on R (Version 4.4). The significance level was set at α = 0.05 for all statistical tests.

#### 2.6.1. Descriptive Statistics

Ranking accuracy was defined as the proportion of cases in which a model’s predicted landmark order matched the TVL-based reference ranking. It was treated as a binary variable, where a prediction was classified as correct only when the model’s estimated anteroposterior order of the three landmarks (ULA, LLA, and Pog′) exactly matched the reference order obtained from the lateral cephalometric TVL analysis. Any deviation from this sequential order, including partial matches, was considered incorrect. No numerical tolerance or margin of error in relative depth values was applied; the evaluation was strictly based on ordinal ranking. This stringent definition was adopted to assess each model’s capacity to produce clinically meaningful and unequivocal anteroposterior relationships, since even minor ranking inconsistencies could alter diagnostic interpretation in orthodontic practice. For DPT-Large, DepthAnything-v2, and ZoeDepth, mean accuracy, standard deviation, and range were calculated across the sample of 82 cases.

#### 2.6.2. Confidence Interval Estimation and Chance Level

Exact binomial tests (Clopper–Pearson method) were used to calculate 95% confidence intervals (CIs) for each model’s observed accuracy. For the three-landmark ranking task, there are 3! = 6 possible permutations, corresponding to a theoretical chance accuracy of 16.7% (1/6) under random prediction. Therefore, the null hypothesis for the exact binomial test was set at *p*_0_ = 0.167, rather than 0.5, to represent the true baseline probability of a correct ranking occurring by chance alone.

#### 2.6.3. Pairwise Model Comparisons (McNemar) and Multiplicity Control

Pairwise model comparisons were performed using the McNemar test to determine whether differences in ranking accuracy between models were statistically significant. This non-parametric test was selected because all three models evaluated the same set of images, producing paired binary outcomes (correct vs. incorrect). Three pairwise comparisons were conducted: (i) DPT-Large vs. DepthAnything-v2, (ii) DPT-Large vs. ZoeDepth, and (iii) DepthAnything-v2 vs. ZoeDepth. The McNemar test statistic follows a chi-square (χ^2^) distribution with one degree of freedom.

To control the family-wise Type I error rate arising from multiple comparisons, the Bonferroni correction was applied (α = 0.0167; 0.05/3). Although the Bonferroni method is recognized as conservative and may increase the risk of Type II errors, it was deliberately adopted to ensure the robustness of statistical significance claims and to minimize the likelihood of false-positive results.

It is acknowledged that the McNemar test assumes independence of classification errors between the two models being compared. Since all models processed the same dataset, partial correlation of errors cannot be excluded, representing a potential limitation of this assumption. Nonetheless, the McNemar test remains the standard approach for assessing marginal homogeneity in paired nominal data and was therefore retained as the primary method for inter-model comparison.

#### 2.6.4. Analysis by Ranking Complexity and Malocclusion Class

To further explore factors that might influence model performance, analyses were conducted according to task complexity and skeletal malocclusion class.

To examine whether accuracy varied according to ranking configuration, images were classified into three categories of complexity: standard, moderate variation, and high variation. A chi-square test of independence was then applied to determine whether the distribution of correct versus incorrect predictions differed significantly across these categories for each model.

Ranking complexity was operationally defined based on the minimum pairwise anteroposterior distance between the three landmarks (ULA, LLA, and Pog′) as measured on the reference cephalometric TVL analysis. Cases in which the minimum inter-landmark distance was less than 2 mm were classified as high complexity, whereas those with distances of 2 mm or greater were classified as low complexity. This threshold was selected according to the reported precision limits of manual cephalometric landmark identification [19,20].

Additionally, to evaluate the potential influence of skeletal malocclusion pattern on depth-estimation accuracy, model performance was analyzed separately for Angle Class I, Class II, and Class III malocclusion groups. Chi-square tests were conducted to determine whether accuracy rates differed significantly across malocclusion classes.

#### 2.6.5. Data Structure and Assumptions

Prior to statistical testing, data were screened for completeness and assumptions. For the McNemar test, (1) paired observations were confirmed, (2) binary accuracy outcomes (correct/incorrect) were validated, and (3) the total sample size (*n* = 82) was deemed sufficient for chi-square approximation. Each subject contributed a single paired observation across models, satisfying the assumption of independence at the subject level. However, because all models processed the same set of images, their prediction errors may be correlated, representing a potential limitation of the independence assumption inherent to McNemar’s test.

#### 2.6.6. Software and Reproducibility

Data were maintained in structured CSV format, and analysis scripts were archived to ensure full reproducibility. Statistical outputs and plots were stored to facilitate verification and future reference.

## 3. Results

The results are presented in seven subsections, covering landmark reliability, model accuracy, error magnitude, pairwise and chance-level analyses, and subgroup evaluations based on ranking complexity and malocclusion class.

### 3.1. Reliability of Landmark Annotation

Intrarater reliability of the manually annotated soft-tissue landmarks was evaluated by calculating Intraclass Correlation Coefficients (ICCs) for coordinates obtained at two independent time points, spaced two weeks apart. All six comparisons demonstrated excellent agreement: ULA_X (ICC = 0.993, 95% CI: 0.990–0.995), ULA_Y (ICC = 0.981, 95% CI: 0.972–0.987), LLA_X (ICC = 0.989, 95% CI: 0.985–0.992), LLA_Y (ICC = 0.986, 95% CI: 0.979–0.991), Pog_X (ICC = 0.985, 95% CI: 0.978–0.990), and Pog_Y (ICC = 0.969, 95% CI: 0.949–0.980). All ICC values exceeded the threshold of 0.90, indicating excellent reliability.

Further support for annotation consistency was provided by low coefficients of variation (CV: 0.74–1.42%) and standard errors of measurement (SEM: 1.44–4.50 pixels). Although Pog_Y exhibited the highest SEM (4.50) and CV (1.42%), these remained within acceptable limits. Overall, the results confirmed that manual annotations were highly reliable and appropriate for use as reference coordinates in subsequent analyses.

### 3.2. Ranking Accuracy Evaluation

Depth-based ranking accuracy revealed a clear performance gradient among the three models (Table 2). DPT-Large achieved the highest accuracy, correctly replicating the ground-truth anteroposterior order in 76 out of 82 cases (92.7%). In contrast, DepthAnything-v2 and ZoeDepth performed substantially worse, with correct rankings in only 8 (9.8%) and 4 (4.9%) cases, respectively.

### 3.3. Error Magnitude Analysis

To further assess the clinical relevance of the observed misclassifications, the magnitude of depth estimation errors was quantified for cases in which the DPT-Large model produced incorrect anteroposterior rankings. Among the six misclassified cases (7.3% of the sample), the mean absolute depth difference between landmarks was 0.88 ± 0.75 (unitless relative depth values). In contrast, correctly ranked cases exhibited a mean absolute depth difference of 2.02 ± 1.31, indicating that errors predominantly occurred when the anteroposterior separation between landmarks was relatively small. Specifically, the minimum depth difference observed among incorrect cases was 0.02, whereas the maximum was 2.69. This pattern suggests that the model’s misclassifications were concentrated in anatomically subtle or borderline cases with low inter-landmark depth contrast, rather than being randomly distributed across the dataset. These findings emphasize that the clinical interpretability of Monocular Depth Estimation depends not only on binary ranking accuracy but also on the relative depth spacing between key facial landmarks.

### 3.4. Pairwise Model Comparisons

Pairwise McNemar tests revealed highly significant differences in ranking performance among the models (Table 3). DPT-Large significantly outperformed both DepthAnything-v2 and ZoeDepth, with large effect sizes (φ = 0.898 and 0.937, respectively), indicating substantial practical advantages. In contrast, no statistically significant difference was observed between DepthAnything-v2 and ZoeDepth (*p* = 0.206), suggesting similarly poor performance levels for these models.

### 3.5. Confidence Interval Analysis

Binomial proportion tests were conducted to determine whether each model’s performance significantly differed from the theoretical chance level of 16.7% (corresponding to one correct ordering among six possible permutations). DPT-Large performed significantly above chance (*p* < 0.001), confirming its reliability. In contrast, both DepthAnything-v2 and ZoeDepth performed significantly below the theoretical chance level (*p* < 0.001), suggesting systematic errors in their anteroposterior predictions (Table 4).

### 3.6. Ranking Pattern Analysis

**Figure 4:**
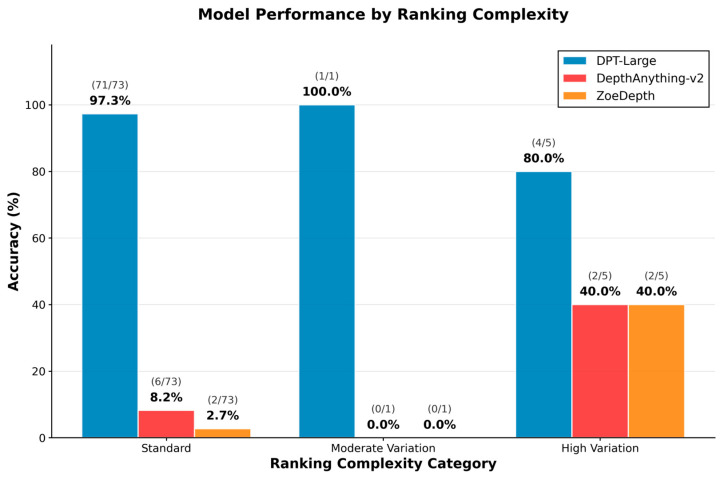
Model performance stratified by ranking complexity: Accuracy across standard, moderate, and high variation ranking types for DPT-Large, DepthAnything-v2, and ZoeDepth.

Performance was further analyzed based on ranking complexity, categorized into standard (ULA > LLA > Pog′), moderate variation, and high variation configurations. As shown in Figure 4, DPT-Large achieved consistently high accuracy across all categories, indicating robustness to anatomical variability. Conversely, DepthAnything-v2 and ZoeDepth exhibited uniformly poor performance, particularly under standard ranking conditions. Chi-square analyses confirmed significant associations between model type and prediction accuracy (χ^2^(4) = 12.0, *p* = 0.017 for correct matches; χ^2^(4) = 20.9, *p* < 0.001 for incorrect matches), highlighting the critical role of model selection in depth-based facial analysis.

### 3.7. Subgroup Analysis by Ranking Complexity and Malocclusion Class

Subgroup analysis revealed that the DPT-Large model’s performance varied slightly according to ranking complexity. Among the 38 cases classified as low complexity (inter-landmark distance ≥2 mm), the model achieved an accuracy of 97.4% (95% CI: 86.2–99.9). In contrast, among the 44 high-complexity cases (inter-landmark distance <2 mm), accuracy was 88.6% (95% CI: 75.4–96.2), representing an 8.7 percentage point reduction. However, this difference did not reach statistical significance (χ^2^ = 1.19, *p* = 0.276), suggesting that although a trend toward reduced performance in high-complexity cases was observed, the model maintained robust accuracy across varying levels of task difficulty.

**Figure 5:**
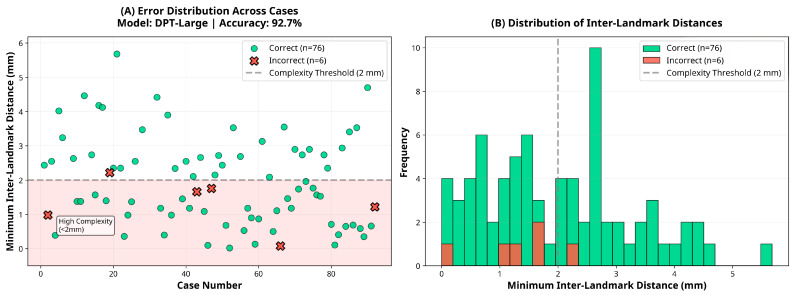
Error and distance distribution for the DPT-Large model.

**Figure 6:**
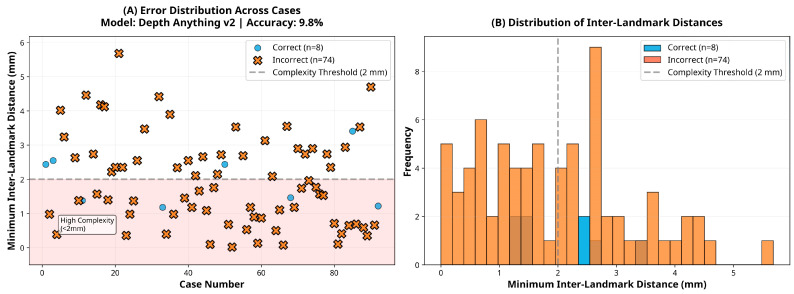
Error and distance distribution for the DepthAnything-v2 model.

**Figure 7:**
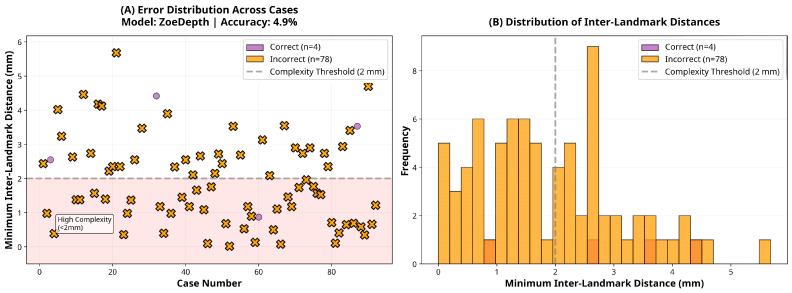
Error and distance distribution for the ZoeDepth model.

With respect to skeletal pattern, the DPT-Large model demonstrated comparable performance across Angle Class I (95.8%, 95% CI: 85.7–99.5, *n* = 48), Class II (89.3%, 95% CI: 71.8–97.7, *n* = 28), and Class III (83.3%, 95% CI: 35.9–99.6, *n* = 6) groups. Chi-square analysis indicated no statistically significant difference in accuracy across malocclusion classes (χ^2^ = 1.95, *p* = 0.377), confirming that the model’s depth estimation performance is robust to variations in sagittal skeletal morphology. These subgroup findings are illustrated in Figure 5, Figure 6 and Figure 7, which show the distribution of inter-landmark distances and classification accuracy across varying levels of task complexity for each model.

## 4. Discussion

The following discussion interprets the main findings considering existing literature, highlighting their methodological, clinical, and theoretical implications.

### 4.1. Methodological Context and Rationale for Monocular Depth Estimation

A depth map encodes the relative distance between the camera sensor and the facial surface on a pixel-by-pixel basis, allowing three-dimensional inference from a single two-dimensional image. Unlike traditional approaches that depend on stereo correspondence or sequential video frames, Monocular Depth Estimation (MDE) algorithms extract depth directly from static visual cues [21,22].

In orthodontics, 3D stereophotogrammetry and facial scanning technologies have gained popularity for soft tissue analysis. However, these methods typically require multi-camera setups, structured light systems, or other specialized hardware—factors that limit clinical accessibility and increase cost. MDE, by contrast, only requires a frontal photograph, making it a low-cost, non-invasive, and widely accessible alternative [14,15,23,24].

The present study introduces a novel diagnostic paradigm by demonstrating that clinically meaningful profile information can be derived from a single frontal image using MDE. To the best of our knowledge, this is the first academic investigation to validate MDE for three-dimensional soft tissue profiling in orthodontics.

The robustness of the reference dataset was supported by excellent reproducibility of landmark annotation, with all ICC values exceeding 0.90 across repeated sessions. This high level of reliability provided a solid methodological foundation for evaluating model performance.

### 4.2. Comparative Model Performance and Architectural Implications

At the core of this investigation were the striking differences in anteroposterior (AP) landmark ranking accuracy across the three evaluated depth estimation models. The transformer-based DPT-Large achieved clinically acceptable accuracy in 76 of 82 cases (92.7%, 95% CI: 84.8–97.3), far exceeding both theoretical chance and clinically relevant thresholds. This superior performance can be attributed to the Vision Transformer (ViT) backbone, which leverages self-attention mechanisms to capture global image context rather than relying solely on localized convolutional kernels [25]. By representing subtle depth gradients across the full facial image, DPT-Large demonstrated robust accuracy (80.0–97.3%) across standard, moderate, and high-complexity anatomical configurations.

In contrast, the CNN-based models DepthAnything-v2 and ZoeDepth exhibited severely impaired performance, with accuracies of only 9.8% and 4.9%, respectively. Both models performed significantly below chance level (*p* < 0.001), reflecting systematic misclassification rather than random variability. This failure likely stems from two key limitations: (1) both models were primarily trained on large-scale general-purpose datasets (e.g., natural scenes, indoor/outdoor environments) rather than high-resolution facial data, and (2) their architectures are optimized for detecting broad depth discontinuities rather than millimetric variations that are critical for orthodontic assessment [26,27]. These models were evaluated using publicly available pre-trained weights without domain-specific fine-tuning to ensure full reproducibility, which may have further limited their adaptation to facial morphology. These shortcomings were particularly evident in standard anteroposterior landmark configurations, where DepthAnything-v2 and ZoeDepth reached accuracies of only 8.2% and 2.7%, respectively.

Collectively, these findings highlight the clear superiority of transformer-based depth estimation architectures for clinically sensitive tasks such as orthodontic profile analysis. At the same time, they underscore the risks of directly transferring general-purpose CNN-based models into medical imaging contexts without domain-specific adaptation. Model architecture and the relevance of training datasets emerge as decisive factors in determining clinical utility.

Beyond orthodontics, Convolutional Neural Networks (CNNs) and multimodal AI models have achieved expert-level diagnostic performance across several medical disciplines. In cardiology, CNN-based algorithms have matched or surpassed clinicians in arrhythmia detection and cardiac imaging interpretation [28,29]. In surgery, deep CNNs have enabled objective skill evaluation in robot-assisted procedures using motion kinematics and 3D ConvNet video frameworks with over 90% accuracy [30,31]. Similarly, in dermatology, CNNs and multimodal large language models such as GPT-4o and Gemini Flash 2.0 have demonstrated strong diagnostic capability in conditions such as hidradenitis suppurativa, acne, and rosacea [32,33]. These cross-disciplinary applications reinforce the potential of Deep Learning architectures to generalize across complex clinical domains, supporting their exploration in orthodontic soft-tissue analysis.

### 4.3. Clinical Interpretation and Diagnostic Relevance

While the DPT-Large model achieved statistically significant superiority over CNN-based alternatives, it is also important to evaluate whether its performance meets clinically acceptable standards. In conventional cephalometric analysis, landmark localization errors of approximately 1–2 mm are generally regarded as tolerable for clinical decision-making [34,35]. Although the unitless depth values generated by Monocular Depth Estimation cannot be directly converted into metric distances, the model’s ability to correctly reproduce the anteroposterior order of landmarks in 92.7% of cases suggests a level of precision that may be sufficient for preliminary sagittal profile assessment, particularly in screening or initial consultation settings. Nevertheless, the clinical acceptability of the model’s errors—especially in cases exhibiting minimal depth contrast—remains to be established. Even a small proportion of misclassifications could lead to inappropriate treatment planning in borderline skeletal cases; thus, aiming for accuracy levels comparable to the ≥95% benchmarks typically required for clinical-grade diagnostic AI systems [36] would help ensure patient safety and clinical reliability.

### 4.4. Limitations and Future Directions

Despite the encouraging findings, several limitations should be acknowledged. The study cohort consisted of 82 patients from a single orthodontic practice, which may limit generalizability. Broader validation using multi-center datasets encompassing multiethnic and morphologically diverse populations will be essential to confirm the robustness of Monocular Depth Estimation (MDE) under varied clinical conditions.

In addition, model performance was evaluated on a single dataset without employing cross-validation or repeated analyses on independent subsets. This methodological constraint suggests that the reported accuracy metrics may be sample-dependent, and their generalizability across different populations remains to be verified. Future studies should incorporate k-fold cross-validation or external validation datasets to assess the stability and reproducibility of the DPT-Large model across varied patient demographics and imaging conditions.

The study sample also exhibited an uneven distribution of malocclusion classes, with Class III cases being markedly underrepresented (*n* = 6, 7.3%). This imbalance reflects the lower global prevalence of Class III malocclusion [16] but may limit the generalizability of the findings to this subgroup. Although no significant difference in model accuracy was observed among classes (χ^2^ = 1.95, *p* = 0.377), the wide confidence interval for Class III (83.3%, 95% CI: 35.9–99.6) indicates uncertainty due to small sample size. Future studies with larger and more balanced cohorts are needed to confirm model reliability across all skeletal patterns, particularly for Class III cases with distinct anteroposterior characteristics.

The present study also did not control for ethnic background, sex, or facial morphology factors known to influence facial geometry and potentially affect depth estimation outcomes. Larger and more demographically diverse cohorts are needed to evaluate model reliability across different ethnic, sex-based, and morphological groups.

A significant limitation of the ordinal ranking approach employed in this study is its reduction in continuous three-dimensional spatial relationships to discrete categorical outcomes. While the method determines the relative anteroposterior order of landmarks, it does not quantify the actual magnitude of these depth differences. In orthodontics, diagnostic and treatment decisions rely on precise millimetric measurements rather than simple ordinal relationships. Consequently, the present methodology cannot replace conventional cephalometric analysis but instead serves as an exploratory framework for evaluating whether Monocular Depth Estimation can capture clinically meaningful sagittal information. Furthermore, conventional quantitative error metrics such as mean absolute error (MAE) or root mean square error (RMSE) were not applied, as the model outputs are unitless and scale-invariant. Future work incorporating depth-to-metric calibration would enable quantitative error assessment and facilitate direct comparison with clinical reference data.

Within the evaluated models, only DPT-Large achieved clinically acceptable accuracy, whereas the CNN-based architectures (DepthAnything-v2 and ZoeDepth) demonstrated lower precision. These differences do not necessarily indicate architectural inferiority but may reflect limited adaptation to orthodontic facial morphology and variations in pre-training data. All models were evaluated using pre-trained weights without domain-specific fine-tuning to ensure full reproducibility and assess out-of-the-box generalization. Fine-tuning with clinically relevant facial datasets or hybrid model designs could substantially improve depth precision and enable a more equitable comparison across architectures. Consequently, the present findings should be interpreted as indicative of each model’s baseline generalization capacity in a zero-shot transfer scenario rather than as definitive evidence of their maximum achievable performance.

### 4.5. Outlook and Clinical Integration

Taken together, these findings indicate that Monocular Depth Estimation (MDE) has the potential to evolve into a clinically supportive adjunct for orthodontic diagnosis rather than a replacement for established radiographic methods. Its future integration into clinical workflows will depend on the development of more precise, quantitatively calibrated models capable of providing true depth measurements and morphometric parameters relevant to soft-tissue evaluation. Subsequent studies should validate these methods across larger and more diverse populations, incorporate a wider range of anatomical landmarks, and assess inter-model consistency to ensure clinical reliability. Fine-tuning transformer-based architectures, such as DPT-Large, on domain-specific facial datasets could further enhance spatial precision and anatomical interpretability. With these advancements, MDE could serve as a complementary diagnostic aid in digital orthodontics—supporting teleconsultation, automated pre-assessment systems, and longitudinal soft-tissue monitoring in everyday practice.

## 5. Conclusions

This study demonstrated the feasibility of applying Monocular Depth Estimation (MDE) to standard frontal facial photographs for orthodontic soft-tissue assessment. The transformer-based DPT-Large model achieved the highest accuracy in reproducing anteroposterior landmark relationships, suggesting that depth information extracted from two-dimensional images may serve as a supportive tool for facial profile evaluation. These findings provide a foundation for future studies exploring the integration of depth-based analysis into digital orthodontic diagnostics.

## Figures and Tables

**Table 1:** Monocular Depth Estimation Model Specifications.

Model	Architecture	Parameters	Pre-Training Dataset	Input Size
DPT-Large	Vision Transformer backbone	≈344 million	MIX 6 (multi-domain depth datasets including NYU Depth v2, KITTI, ScanNet, and ETH3D)	384 × 384
DepthAnything-v2	CNN-based encoder–decoder with feature fusion	≈97 million	Hypersim, Virtual KITTI, Taskonomy	518 × 518
ZoeDepth	CNN-based metric depth network	≈87 million	NYU Depth v2, KITTI, DIML	384 × 384

**Table 2:** Ranking accuracy of depth estimation models.

Model	Correct/Total	Accuracy (%) *	95% CI
DPT-Large	76/82	92.7	84.8–97.3
DepthAnything-v2	8/82	9.8	4.3–18.3
ZoeDepth	4/82	4.9	1.3–12.0

* Accuracy defined as the proportion of cases in which the model’s predicted ranking matched the ground truth (TVL-based reference).

**Table 3:** Pairwise model comparisons (McNemar tests).

Comparison	χ^2^	df	*p*-Value	φ	Effect Size
DPT-Large vs. DepthAnything-v2	66.1	1	<0.001	0.898	Large
DPT-Large vs. ZoeDepth	72.0	1	<0.001	0.937	Large
DepthAnything-v2 vs. ZoeDepth	1.60	1	0.206	0.140	Small

Note: McNemar tests were used for paired binary comparisons. Effect sizes are reported as phi coefficients. Bonferroni-corrected threshold for statistical significance: *p* < 0.0167.

**Table 4:** Binomial proportion test results.

Model	Proportion	*p*-Value	95% CI	Performance vs. Chance
DPT-Large	0.927	<0.001	0.848–0.973	Significantly above
DepthAnything-v2	0.098	<0.001	0.043–0.183	Significantly below
ZoeDepth	0.049	<0.001	0.013–0.120	Significantly below

Note: Exact binomial tests were conducted against the null hypothesis (H_0_: proportion = 0.167, representing the theoretical chance accuracy for six possible landmark orderings).

## Data Availability

The data presented in this study are available on reasonable request from the corresponding author. The data are not publicly available due to privacy and ethical restrictions related to patient confidentiality.

## Abbreviations

The following abbreviations are used in this manuscript:

2D	Two-Dimensional
3D	Three-Dimensional
AI	Artificial Intelligence
AP	Anteroposterior
CNN	Convolutional Neural Network
DPT	Dense Prediction Transformer
GPU	Graphics Processing Unit
ICC	Intraclass Correlation Coefficient
LLA	Lower Lip Anterior
MDE	Monocular Depth Estimation
SD	Standard Deviation
TVL	True Vertical Line
ULA	Upper Lip Anterior
